# The Challenge of Reorganizing Rehabilitation Services at the Time of COVID-19 Pandemic: A New Digital and Artificial Intelligence Platform to Support Team Work in Planning and Delivering Safe and High Quality Care

**DOI:** 10.3389/fneur.2021.643251

**Published:** 2021-04-29

**Authors:** Alessia Saverino, Paola Baiardi, Giuseppe Galata, Gloria Pedemonte, Claudio Vassallo, Caterina Pistarini

**Affiliations:** ^1^Rehabilitation Unit, Istituti Clinici Scientifici Maugeri, Genoa, Italy; ^2^Scientific Direction, Istituti Clinici Scientifici Maugeri, Istituti di Ricovero e Cura a Carattere Scientifico (IRCCS), Pavia, Italy; ^3^SurgiQ Srl, Genoa, Italy; ^4^Department of Neurorehabilitation, Istituti Clinici Scientifici Maugeri, Istituti di Ricovero e Cura a Carattere Scientifico (IRCCS), Pavia, Italy

**Keywords:** COVID-19, centers rehabilitation, goal-directed therapy, staffing and scheduling, artificial intelligence

## Abstract

**Introduction:** The COVID-19 pandemic has posed great challenges in inpatient rehabilitation services, not only to implement the preventative measures to avoid the spreading of the virus in a highly interactive, multidisciplinary setting but also to create a rehabilitation pathway for post-COVID-19 patients. The aim of this retrospective study was to describe the role of a digital and artificial intelligence platform (DAIP) in facilitating the implementation of changes in a rehabilitation service during the COVID-19 pandemic.

**Materials and Methods:** We gathered qualitative and quantitative descriptors of the DAIP, including measures to assess its efficiency in scheduling therapy sessions, and staff satisfaction using two simple numeric rating scales and the System Usability Scale. We describe how the volume of activity and the quality of care of our rehabilitation service have changed when the DAIP was implemented by comparing the pre-COVID-19 and the pandemic periods for patients' [sex, age, co-morbidities, diagnosis, and Functional Independence Measure (FIM) gain] and service's (bed occupancy, patients' length of stay, and staff capacity) characteristics.

**Results:** Bed occupancy and the impact of rehabilitation on patients' outcome remained stable between the two periods. The DAIP provided a qualitative support for goal setting from remote; 95% of the planned sessions were delivered; the time for scheduling and registering sessions dropped by 50%. Staff satisfaction was about 70% for the easiness and 60% for the usefulness, and the mean “usability” score was close to the cut off for sufficient usability (mean score 65 where 68 is the cut off).

**Conclusion:** By applying the DAIP to rehabilitation treatment, it was shown that the management of rehabilitation can be efficiently performed even in the COVID-19 pandemic. Staff satisfaction reflected a good acceptance of the changes considering the turbulent changes and the stress burden occurring at the time of the pandemic.

## Introduction

The global COVID-19 pandemic has determined a great pressure on medical resources worldwide and transformed the organization of health services particularly in countries where the virus spread has been more intense during the two waves of the outbreak. Understandingly, the priority of health care reorganization so far has been on acute care services rather than on the post-acute rehabilitation (1).

Rehabilitation services had to face the challenge of proving the usual care under the increased pressure from the acute sites and of developing dedicated rehabilitation pathways for COVID-19 survivors (2, 3). In fact, the rehabilitation pathway starts from the early acute care and continues in the post-acute and long- term phases based on the complexity of patients' needs and as an integral part of patient's management from hospital to the community setting. Inpatient rehabilitation services had to introduce dedicated pathways for COVID-19 survivors in the post-acute phase, remaining with the more severe physical, emotional, and cognitive sequelae of the viral infection. Although, initially, these pathways have been modeled on the principle of respiratory rehabilitation in COPD and post-ITU syndrome (4), evidences are growing supporting the efficacy of rehabilitation interventions more specifically tailored for post-COVID-19 patients (5–9).

Furthermore, in order to implement COVID-19-related safety measures and to copy with a potentially reduced staff capacity, rehabilitation services have considerably modified their activity, struggling to maintain the same level of service both in terms of the capacity and the quality of care delivered (2, 10). Specific protocols of treatment have been put in place for managing the risks related to the spread of the infection, such as the regulation of personal protective equipment (PPE), disinfection and sterilization protocols, and social distancing between staff members and between patients during therapy sessions, which had to be adapted to the very specific characteristics of rehabilitation settings.

First of all, rehabilitation services provide a multi-disciplinary treatment. This means that many different professionals (including doctors, therapists, nursing staff, and psychologists) interact with single patients and as a team on a daily basis. Group therapy (more patients supervised by one or more therapists) is also an integral part of rehabilitation protocols. The team model itself is based on a highly interactive and coordinated work with regular meetings to set patient-centered goals and discuss patients' progression, as by the definition of an individual rehabilitation program (IRP). Secondly, a direct and prolonged contact between patients and operators is expected, including hand-on or close-distance interventions to support patients in activities such as assisted exercises and ADLs (like dressing, toileting, and feeding) or mobility (like transfers or assisted walking). Furthermore, the use of facilities, equipment, and devices deserves attention in terms of social distancing and disinfection.

The Italian Society of Neurological Rehabilitation (SIRN) has made recommendations (2) to guide activities in rehabilitation units, including the suspension of all meeting activities, replaced by telephone or email and the delivery of rehabilitation activities in patients' rooms whenever possible or, in case of activities taking place in the gym, maintaining at least a 2-m distance between patients.

The change of patients' flow and characteristics together with the safety measures to be introduced in a very particular setting have all posed a particularly challenging reorganizational task for rehabilitation services.

Digitalization and artificial intelligence (AI) systems have shown some promising solutions not only to battle the virus (11) but also to face the organizational difficulties in delivering health care at the time of the outbreak, including systems to prevent the spread of COVID-19 (12), to generate knowledge about the efficacy of certain drug treatments (13), to process COVID-19 related images (14), or to manage the backlog of surgical waiting lists (15). However, there is no digital or AI-based application described in the literature to support rehabilitation services reorganization during the pandemic.

This paper describes how the adoption of a digitalization and artificial intelligence platform (DAIP) could facilitate the implementation of changes in a rehabilitation service during the COVID-19 pandemic while maintaining high-quality standard of care.

In particular, we describe how this DAIP (1) could support the communication between staff for sharing patients' assessment, goal setting, and action plan from remote, (2) could optimize the allocation of therapy sessions (when, where, and how many patients and therapists at the same time), and (3) could be accepted by the staff.

## Materials and Methods

### Project Design

This is a retrospective observational study.

### Setting and Inclusion Criteria

This project took place in the 67-bed ICS Maugeri Rehabilitation Unit in Genoa admitting patients discharged from local acute hospital units requiring multidisciplinary rehabilitation to people affected by neurological and musculoskeletal disorders or, more recently, to COVID-19 survivors remaining with physical, cognitive, and emotional difficulties. The rehabilitation team includes rehabilitation medicine physicians, nursing staff, psychologists (PSY), and therapists. The therapy disciplines range from physiotherapy (PT), occupational therapy (OT), speech and language therapy (SLT), and psychology. The treatment is delivered either in patients' rooms or in therapy-dedicated spaces including one main gym; a second small gym; and single rooms set up for OT, SLT, and PSY.

Data were gathered during two observation periods: May to November 2019 (pre-COVID-19) and May to November 2020 (during the COVID-19 pandemic following the DAIP implementation).

For these two periods, all patients admitted for rehabilitation to our service and the related team activities were included.

### Data Availability

The data associated with the paper are not publicly available but can be obtained from the corresponding author on reasonable request.

### Ethics

All data in this study were collected retrospectively and derived from data and outcome measures used in the routine clinical practice and service evaluation of our rehabilitation service. Patients admitted to ICS Maugeri gave their written consent to the management of their confidential data. The ICS Maugeri informed consent for the treatment of confidential data includes their use for research purposes and it is available to the public online (16).

### Digital and Artificial Intelligence Platform

**Figure 1:**
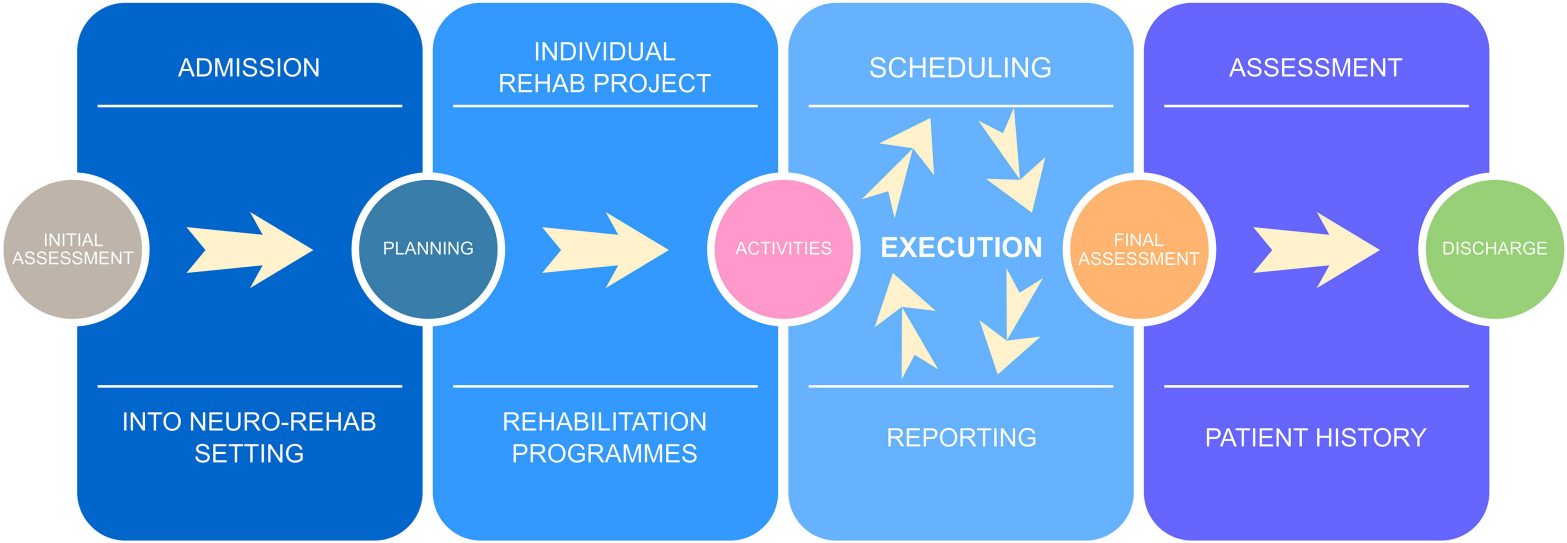
Key steps of the rehabilitation treatment.

The DAIP, which was newly developed and introduced from May 2020, is made of two main software (Priamo and Q-Rehab), which represent the platform for the management of the two key sequential steps of the rehabilitation pathway from the definition of the IRP to its delivery, by scheduling and recording of therapy activities, as represented in Figure 1. The additional value of the DAIP during the COVID-19 pandemic was to support the communication between staff for sharing patients' assessment, goal setting, and action plan from remote (Priamo) and to optimize the allocation of therapy sessions (Q-Rehab) respecting COVID-19-related safety measures when establishing when, where, and how many patients and therapists at the same time.

Priamo was developed by a partnership between the Biomedical Computering System and ICS Maugeri, which started in September 2019. Staff training in our rehabilitation service included a half-day introduction course for all staff, three or four one-to-one 1-h training sessions to a couple of champions selected among doctors and therapists, and monthly drop-in sessions for the first 5 months. Training was arranged and delivered by a dedicated rehabilitation processes team by Maugeri and supported by peer-to-peer support by the champions.

This software is an interactive multidisciplinary platform, allowing patients' evaluation based on the bio-psycho-social model of the International Classification of Functioning framework (17) and the assignment of coherent goals (18–20) for therapy treatment that are established collaboratively by the team. This creates the structure of the IRP. The platform suggests goal areas based on the selection of patients' diagnosis (a list of the main musculoskeletal and neurological pathologies and post-COVID-19) and on the specific impairments and functional limitations that have been identified. Although the system encourages to go through pre-selected lists of functional limitations and matched goals, it allows the flexibility to set up a highly individualized program.

Following the first week of in-depth multidisciplinary assessment and goal negotiation with the patients, each therapist can suggest new goals or define them more in details sharing the same platform. Priamo also includes a weekly goal revision, in the form of an open space where the different members of the treatment team can update the progression or barriers on goals and suggest actions, which are then summarized by the supervising doctor. Priamo is thought to be a support tool for the weekly multidisciplinary meeting; however, during the pandemic, as regular team meetings had to stop, it was completed from remote.

The introduction of the post-COVID-19 rehabilitation meant generating a new dedicated pathway available on Priamo for these patients, based on the best scientific knowledge and expert consensus (3, 4, 21) available so far. Therapy treatment ranges from PT to improve exercise tolerance, endurance, balance, and respiratory function; OT to improve independence in ADL and access to equipment (22); SLT for improving swallowing (23); and psychology to address the psychological and cognitive needs (24–26) of these patients.

The second software (Q-Rehab) is an application based on artificial intelligence algorithms to process and schedule rehabilitation activities into a daily timetable. Q-Rehab was born in January 2019 from a collaboration between Surgi-Q, an EIT Health Headstart start-up, and ICS Maugeri, aiming to develop a novel approach to plan such a complex and multi-professional set of treatments as neurorehabilitation requires. Two therapist coordinators from our rehabilitation service have worked alongside with the software engineers for the development of the software and the provision of staff training. This included one introduction session for all staff, followed by one-to-one 1-h induction for all therapists.

Besides the AI module, the application currently includes a registration and authentication process integrated with ICS Maugeri's overall system, a local database for storing and retrieving the scheduling data, and a graphical user interface to easily visualize and modify patients' timetables and to insert the operator and patient data necessary to the scheduling process. The automated timetable can be manually modified by the therapist coordinator based on specific needs using the drag and drop function enabled in the graphical interface. The AI module uses a programming paradigm developed in the field of non-monotonic reasoning and logic programming. The algorithms operate on constraints and preferences, which have been identified by a joint work between the software engineers and the therapists of the team. The constraints take into account governance quality standards (i.e., a minimum time of daily therapy treatment, a fair number of patients assigned to a therapist based on his/her working hours, etc.) that are rehabilitation-specific as well as COVID-19-related safety recommendations. These included a maximum number of patients per space (i.e., to guarantee social distancing in the gym and avoid sharing of equipment) or patients to be treated in their own room (i.e., requiring isolation and to be treated with appropriate PPE). The algorithms allow taking into account some preferences, once the constraints are met, such as the preferred time by patients and the inclusion of as many supervised sessions as possible.

**Figure 2:**
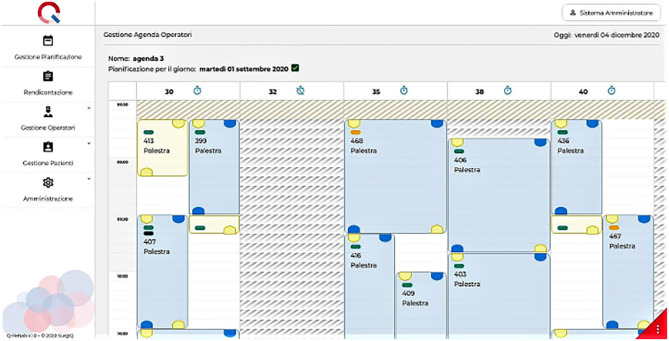
Daily agenda as provided by Q-Rehab. Each column corresponds to a therapist's schedule, with planned sessions represented by colored rectangles (blue for one-to-one sessions and yellow for supervised sessions).

It is the role of three therapy coordinators to input and fill in the data into the system to create a daily dashboard available for staff and patients (Figure 2). Each treating therapist has to confirm if the planned activities have taken place; these are registered by the system and can be displayed as a summary of planned and delivered activities.

### Outcome Measures

We collected patients' general characteristics, such as sex, age, and length of stay (LOS). Clinical measures included the diagnosis (neurological, musculoskeletal, or post-COVID-19), the number of comorbidities, the level of disability (Functional Independence Measure, FIM, at admission and discharge), and the impact of rehabilitation (FIM gain) (27).

We also collected measures of the volume of activity of our service as well as the number of hours worked by the staff members of the different therapy disciplines.

For the period May–November 2020, we tested the efficiency and staff satisfaction for the DAIP.

Three variables of efficiency have been taken into account to evaluate the usefulness of the DAIP.

The first one was the total minutes of therapy sessions delivered to patients either by individualized (“one-to-one” sessions) or supervised practice (one therapist supervising more patients at the same time) and how this has changed month by month (coefficient of variation, defined as the ratio of the standard deviation to the mean).

The second was the ratio between the minutes of planned and delivered sessions, as reported in the database entries and directly calculated by the Q-Rehab software, which is a component of the DAIP. The latter is the time needed to schedule therapy sessions using the Q-Rehab software in comparison to manual scheduling. This variable was calculated as the average time per day spent by 15 therapists during two consecutive weeks using the manual scheduling method (in November 2019) and the Q-Rehab method (in June 2020). The time to schedule therapy sessions took into account the total minutes utilized from registering the data of the patients until the production of the final dashboard.

Considering the impact of the DAIP on staff, either in terms of their routine work and mindset, we measured staff satisfaction for the DAIP. In November 2020, we administered to all staff a 0–10 numerical rating scale (NRS), asking to rate the easiness and the usefulness of the DAIP (where 0 was not at all satisfied and 10 was 100% satisfied) and the System Usability Scale (SUS) (28). The SUS was originally created by John Brooke in 1986. It provides a “quick and dirty” reliable tool for measuring the usability of a wide variety of products and services, including hardware, software, mobile devices, websites, and applications. It consists of a 10-item questionnaire with five response options ranging from “strongly agree” to “strongly disagree;” the responses can be converted into a total score, indicating a not sufficient (0–68), sufficient (68–74), good (74–80), or excellent (>80) usability.

### Statistical Analysis

Data are summarized as means and standard deviations (SD) and frequencies and percentages for quantitative and qualitative variables, respectively. Ordinal data are presented as medians and interquartile range (IR). Comparisons between figures observed in 2019 and 2020 were assessed by means of unpaired Student's *t*-test or chi-square test, as appropriate. Non-parametric statistics, namely, Mann–Whitney *U*-test, was applied to test the differences in the number of comorbidities between patients hospitalized in the 2 years. Missing data occurred in <1% of FIM evaluations; thus, no imputation of missing data was considered.

Statistical significance was set at 0.05. SPSS statistical software was used to perform the analyses.

## Results

### Patient and Service Characteristics Pre-COVID and During COVID-19 Pandemic

Patient characteristics in the two periods, pre-COVID-19 and during COVID-19 pandemic, are displayed in Table 1. In 2019, 34.8% of patients were admitted with a neurological diagnosis [of which 43% were stroke, 16% spinal cord injury (SCI), 14% Parkinson's disease, 9% multiple sclerosis (MS), and 18% other diagnosis] and 65.2% with a musculoskeletal diagnosis (of which 45% were fractures and polytrauma, 27% knee and 21% hip arthroplasty, and 7% others). In 2020, 41.6% were admitted with a neurological diagnosis (of which 60% were stroke, 13% SCI, 5% traumatic brain injury, 2.5% MS, and 19.5% others), 51.5% with a musculoskeletal diagnosis (of which 47% were fractures and polytrauma, 26% hip and 19% knee arthroplasty, and 8% others), and 7.2% post-COVID-19.

**Table 1:** Comparison of patients' characteristics in the two periods.

	**May–November 2019**	**May–November 2020**	***p*-value**
Total of patients	351	262	<0.001
•Neurological, *N* (%)	122 (34.8)	117 (41.6)	
•Musculoskeletal, *N* (%)	229 (65.2)	145 (51.5)	
•Post-COVID-19, *N* (%)	0	19 (7.2)	
Males, *N* (%)	122 (65.2)	110 (42.0)	0.07
Females, *N* (%)	229 (34.8)	152 (58.0)	
Age, mean (SD)	73.2 (11.4)	73.2 (11.9)	0.97
FIM admission, mean (SD)	79.0 (21.3)	72.8 (20.4)	<0.001
FIM discharge, mean (SD)	98.1 (23.7)	95.5 (23.8)	0.18
FIM gain, mean (SD)	19.1 (10.7)	22.5 (12.1)	<0.001
*N* of comorbidities, median (RI)	3 (1)	3 (1)	0.37
LOS	26.7 (16.3)	32.4 (19.7)	<0.001

*FIM, Functional Independence Measure; LOS, length of stay. FIM gain = FIM discharge – FIM admission*.

**Figure 3:**
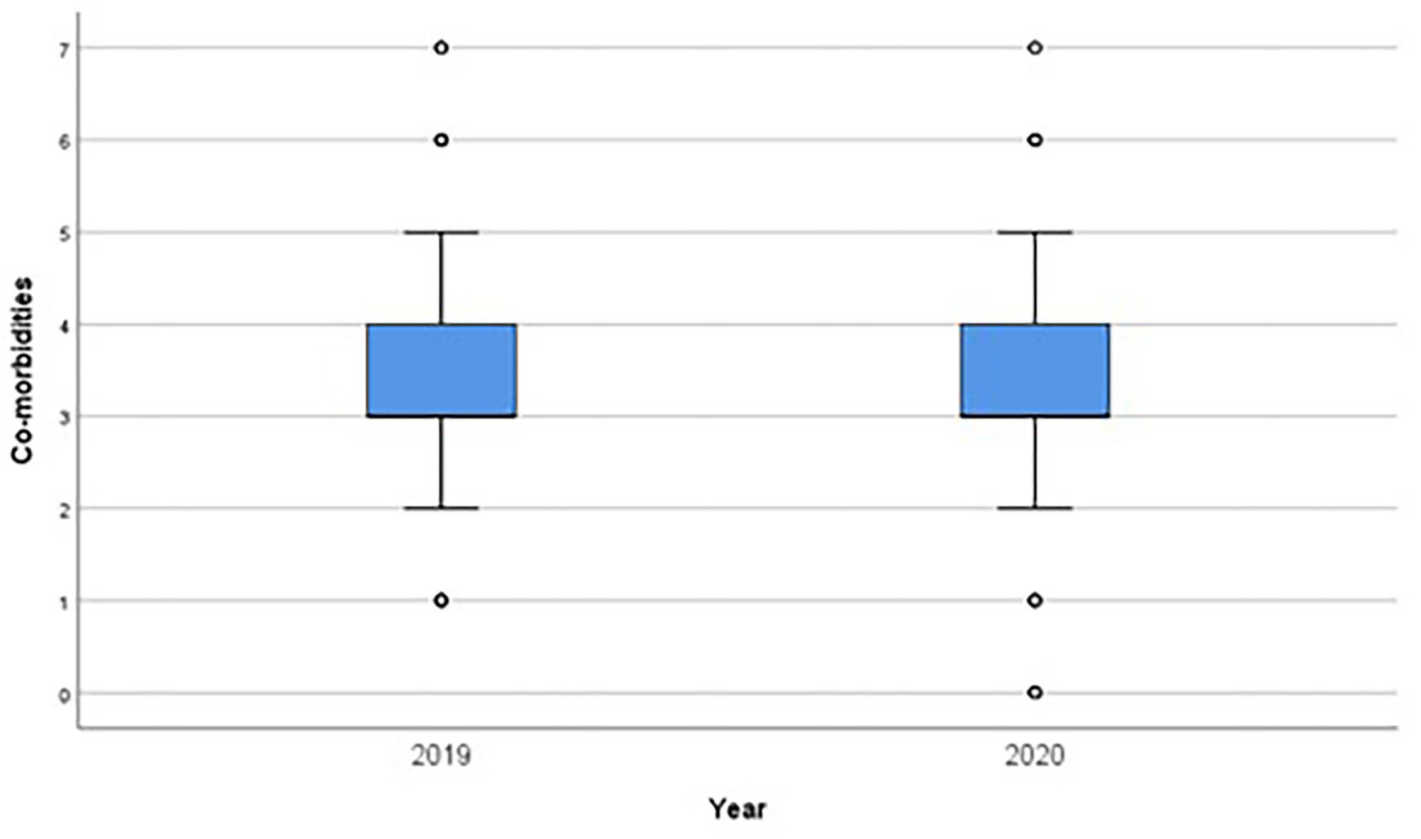
Number of comorbidities in 2019 and 2020.

There was no statistical difference between the two periods for sex, age, and number of comorbidities (Figure 3). Bed occupancy was >90% in the two periods, achieving the target for our service.

**Figure 4:**
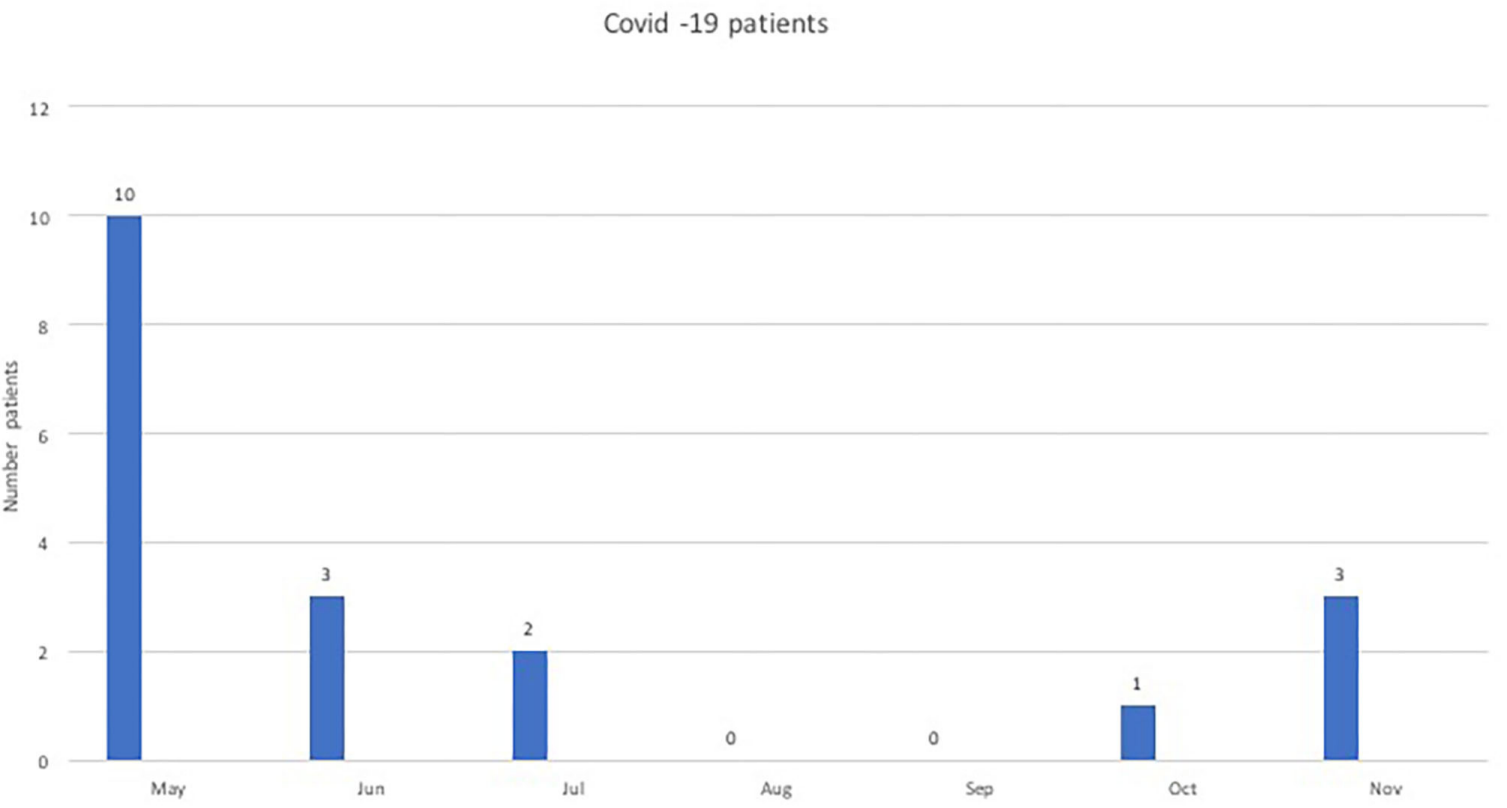
Post-COVID19 patients admitted for rehabilitation.

In the period of May–November 2020, we admitted 19 patients for post-COVID-19 rehabilitation. The flow of post-COVID-19 patients into our rehabilitation service reflected the post-acute timing of the two waves of the outbreak (Figure 4). During this period, the total number of patients admitted was lower, the mean LOS was longer, the mean admission FIM was lower, and the mean FIM gain was larger. Although there was no statistically significant difference in staff capacity, the total number of hours for nursing, PT, and SLT was less in May–November 2020, while it increased for OT and psychology (Table 2).

**Table 2:** Volume of activity and staff capacity in the two periods.

	**May–Nov 2019**	**May–Nov 2020**	***p*-value**
**OBD** ***service target***	**>90%**	**>90%**	
Hours nursing, average/month (SD)	3,357 (142)	3,307 (136)	0.52
Hours medical, average/month (SD)	1,327 (94)	1,354 (83)	0.57
Hours PT, average/month (SD)	1,783 (169)	1,669 (116)	0.17
Hours OT, average/month (SD)	49 (15)	71 (12)	0.01
Hours SLT, average/month (SD)	223 (27)	209 (41)	0.30
Hours PSY, average/month (SD)	379 (76)	412 (57)	0.37

*OBD, occupied bed days; PT, physiotherapy; OT, occupational therapy; SLT, speech and language therapy; PSY, psychology; SW, social work*.

### Allocation of Therapy Sessions by Q-Rehab

**Figure 5:**
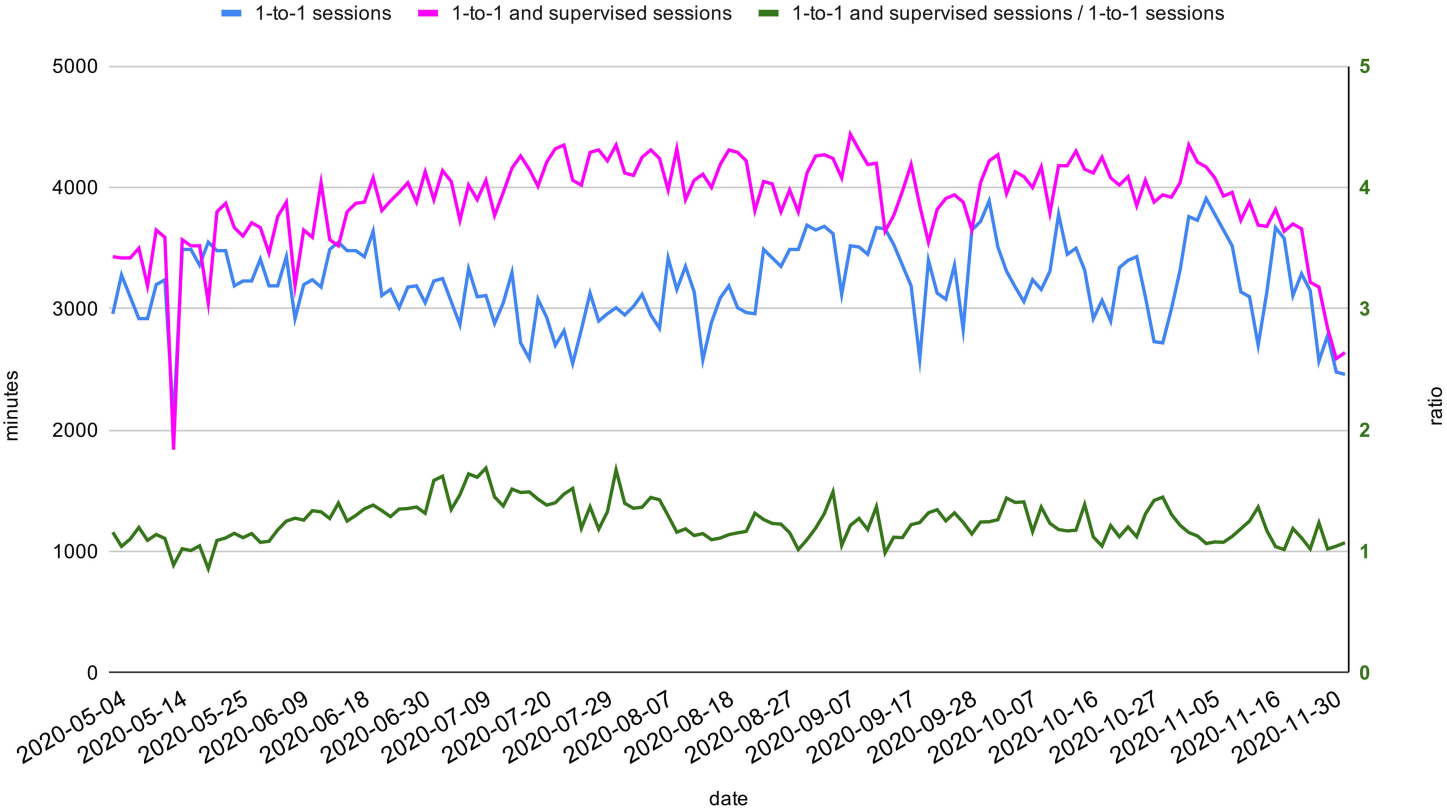
Total daily minutes of therapy sessions. Total daily minutes of one-to-one and minutes of all sessions (both one-to-one and supervised) between May 2020 and November 2020 (light blue and dark blue on the left axis). The ratio between the two aforementioned quantities (pink) is represented on the right axis.

**Figure 6:**
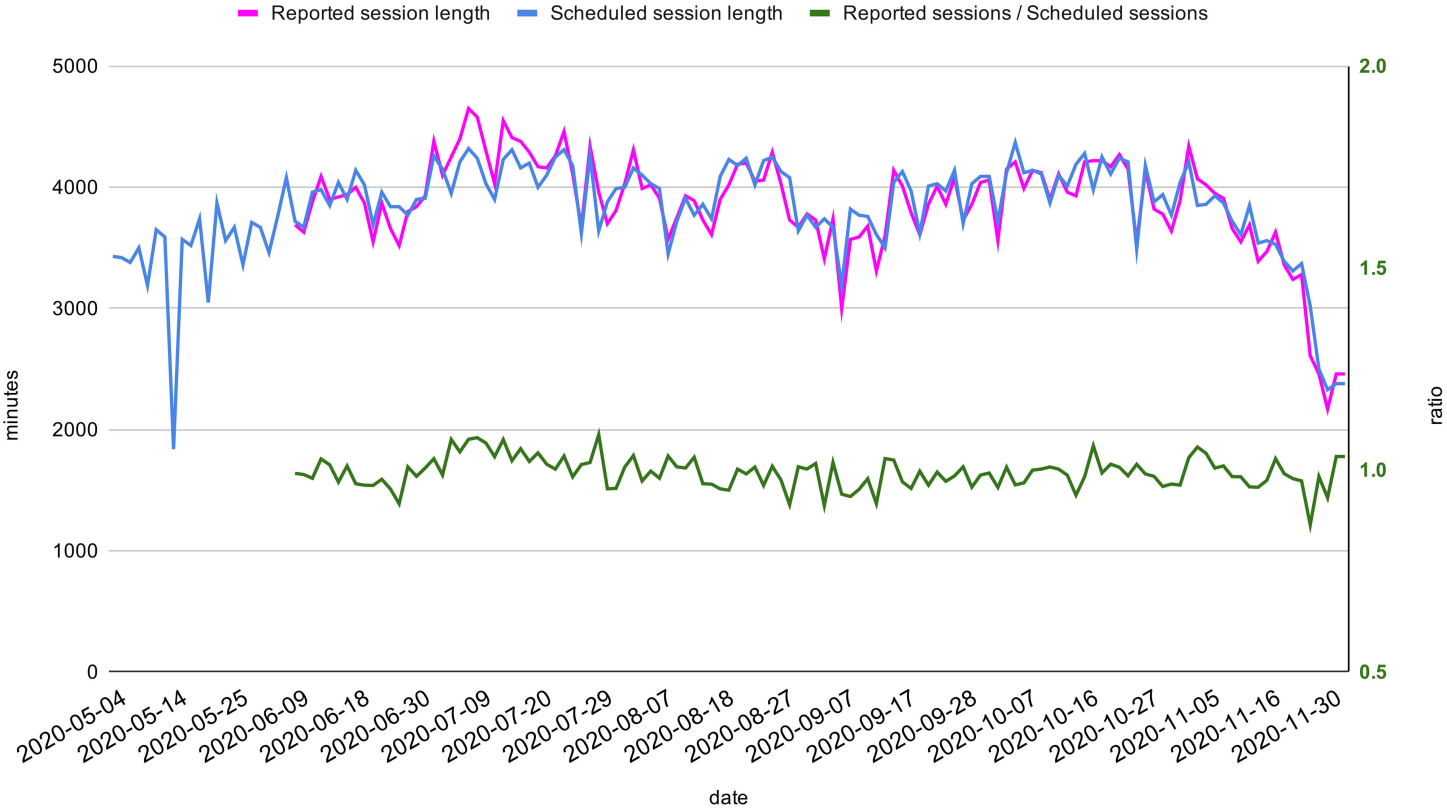
Planned and reported sessions. Total daily minutes of planned and reported sessions between May 2020 and November 2020 (respectively pink and blue, on the left axis). The reporting activity has only started in June. The ratio between minutes of reported and planned sessions (green) is displayed on the right axis.

The total daily minutes of delivered therapy sessions remained fairly constant (mean 3,899 min; SD 371) throughout the period of the COVID-19 pandemic, even considering an initial drop in May 2020. A slight increase of the ratio between supervised and “one-to-one” sessions in July and August 2020 is notable in Figure 5. As shown in Figure 6, there has not been a considerable discrepancy between minutes of reported and planned sessions. In particular, the ratio between these two quantities has been >0.95 for the 95% of the considered time span.

**Figure 7:**
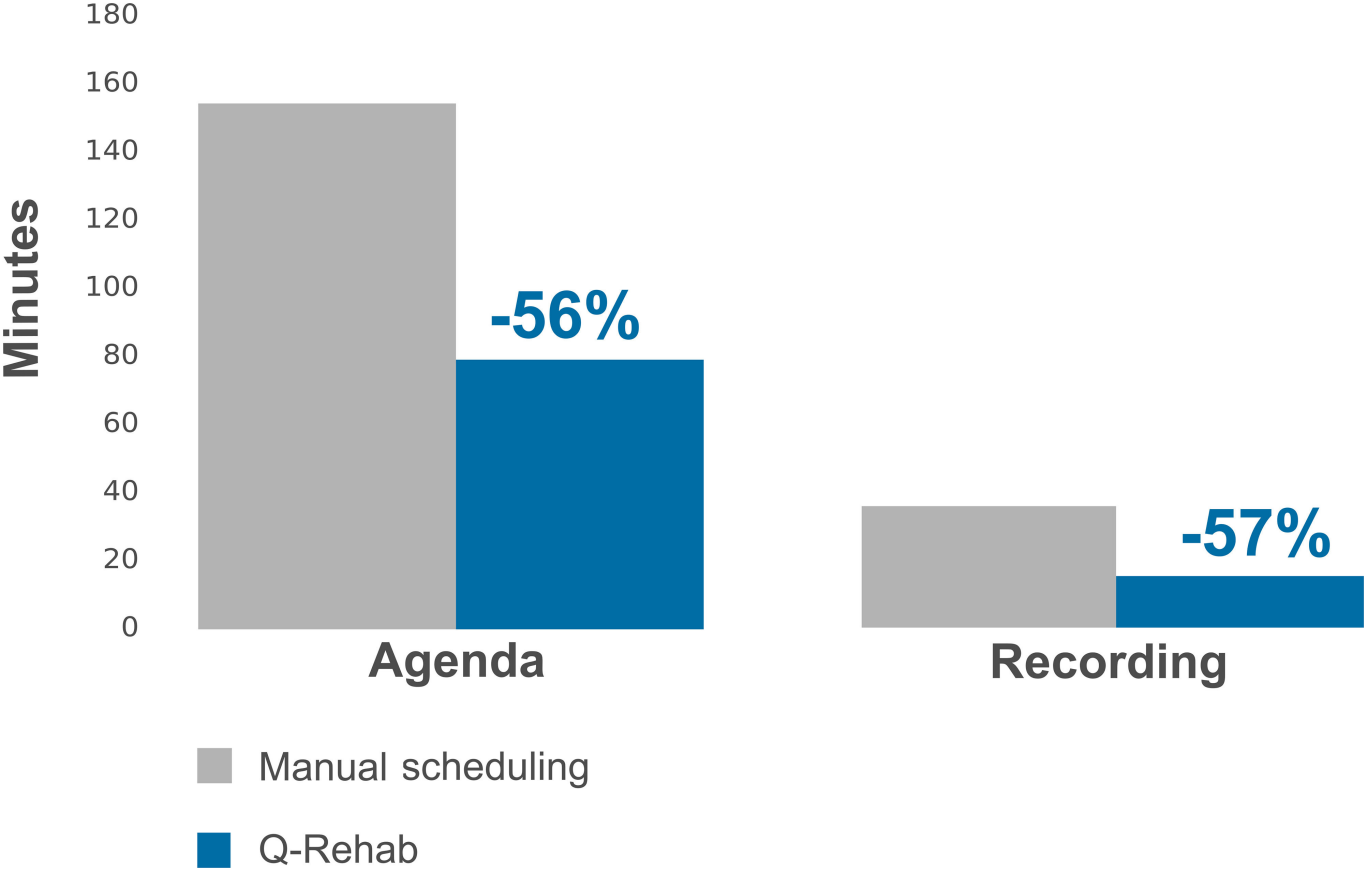
Time saved in scheduling and recording therapy sessions.

The mean time per day needed to schedule and record patient therapy sessions by 15 therapists using the manual method was 153 and 35 min, respectively, for scheduling and recording, while using Q-Rehab, it dropped to 78 and 15 min, respectively. The time percentage saved by the software is represented in Figure 7.

### Staff Acceptance of the DAIP

The two NRS and the SUS were administered to all the operators directly involved in the use of the two software (medical staff for Priamo; therapists and psychologists for both Priamo and Q-Rehab) and returned by four doctors (66%), eight PT (57%), two PSY (66%), two SLT (100%), and zero OT (0%). The mean NRS score (SD) for the easiness/usefulness of Priamo was, respectively, 7.1 (2.4) and 6.25 (1.4), while the mean NRS score (SD) for the easiness/usefulness of Q-Rehab was, respectively, 7.25 (1.5) and 5.9 (2.6) (Table 3). The SUS mean total score (SD) was 65.6 (13) for Priamo and 65.8 (11.9) for Q-Rehab.

**Table 3:** Staff satisfaction in the use of the DAIP.

	**NRS (0–10) easiness Mean (SD)**	**NRS (0–10) usefulnessMean (SD)**	**SUS total score Mean (SD)**
Priamo^a^	7.1 (2.4)	6.25 (1.4)	65.6 (13)
Q-Rehab^b^	7.25 (1.5)	5.9 (2.6)	65.8 (11.9)

a *Scored by four doctors, eight PT, two PSY, and two SLT*.

b *Scored by eight PT, two PSY, and two SLT*.

## Discussion

Our rehabilitation service faced a challenging time to remodel itself and adapt to the new patients and service needs during the COVID-19 pandemic. New governance rules and operational policies to protect patients and staff against the spread of the virus had to be implemented and put in place from the start of the pandemic. Moreover, COVID-19 survivors with high rehabilitation needs started to be admitted to our rehabilitation service in May 2020, for whom an *ad hoc* rehabilitation pathway had to be set up, based on the newly developed international expert recommendations and guidelines (3–10). Progressive learning and changes continue to occur to face the rehabilitation pandemic as unfortunately the virus has not been defeated yet (1, 29, 30).

This paper describes how the adoption of a DAIP helped our rehabilitation service to maintain the high-quality level of care provided by our service (patient outcome) by offering the infrastructure for the team planning and actioning of the IRP while accommodating for the change in the type of the patients being admitted and the new COVID-19-related safety measures.

The DAIP and its implementation have been prepared for a long time before the pandemic, aiming for a quality improvement of our service. The DAIP's additional value during the pandemic was to implement the post-COVID-19 rehabilitation pathway and to schedule and register therapy sessions automatically in a safe and efficient way.

We have shown that the average FIM gain, which is a standard commonly used index of rehabilitation impact applicable to patients across different diagnoses (27), remained at least similar in the two periods, as the statistical difference is likely not clinically relevant (31). Although this is a rather rough measure of rehabilitation quality, it reflects the core outcome variable of the team's work, i.e., the functional change achieved by patients.

We have also described how the different actors of the scene have changed in the new context, i.e., the type of patients being admitted and a more multi-disciplinary treatment team. In particular, we observed a reduced number of admission of patients with musculoskeletal disorders in 2020, related to the discontinuation of elective surgery during the pandemic. Although they may share a similar medical complexity (similar number of comorbidities) with patients admitted with musculoskeletal disorders, patients with neurological disorders or post-COVID-19 are likely more complex in terms of therapy needs, due to the co-existence of physical and cognitive problems, and generally require a longer stay.

Although not statistically significant, some changes took place in the total hours worked by the different staff disciplines, i.e., less hours for nursing, SLT, and PT and more hours for PSY and OT. These changes may reflect a drop of hours due to sick leave for certain therapy disciplines but at the same time show a progressive shift toward a more multi-disciplinary model of rehabilitation.

We have described the qualitative and quantitative contributions that Priamo and Q-Rehab provided.

The qualitative impact of Priamo was to offer a digital room where the team could virtually meet to formulate the IRP and review patients' progression on goals, thus allowing a multi-disciplinary interaction and team goal setting from remote. This reduced the time of direct staff interaction in an apparently easy-to-use intuitive fashion.

Even if we have not quantified the impact of Priamo, for example, on the type of goals being set or their achievement, we have described how it works and staff-related satisfaction. The structure of Priamo is based on the ICF framework for the definition of the IRP following established rehabilitation pathways and producing a consistent goal setting across patients and sound clinical records. Priamo could not substitute the value of multidisciplinary meetings made of people interacting in a physical room, and further communication was still occurring by email, phone, and direct one-to-one talking.

Staff feedback on Priamo showed that it proved to be relatively easy to use (about 70% satisfaction on the easiness) and its “usability” was close to sufficient (SUS mean total score was 65.6, cut-off for usability is 68) with about 65% satisfaction on the usefulness. We suggest that some of the concerns might have come from the limitation of working from remote, reducing the team's direct interaction. An alternative or additional option could have been arranging multi-disciplinary meetings and goal setting via video conference; however, this option was perceived by the team as an excessive burden on staff members, not allowing a real life-like interaction, with expected technical problems and difficulties in updating electronic clinical records in real time.

We tested how Q-Rehab could optimize the allocation of therapy sessions, by measuring its efficiency in different ways. Our results show that the total time of the sessions delivered to patients remained stable during the pandemic and meeting the quality standards for the service.

The slight drop of the total session time in May (Figure 5) may suggest the need for an initial adjustment to the new situation, while the “one-to-one” sessions' drop (Figure 5) in July and August is more likely related to staff annual leaves during summer, which was well-compensated by the increase of supervised sessions. The high level of adherence to the scheduled agenda demonstrates the excellent reliability and efficiency of the agenda generated by Q-Rehab.

A significant finding was the halving of the scheduling and activity recording time using the Q-Rehab software. The time saved by the therapists in scheduling and recording their activity could have been productively used for wearing PPE, disinfection of equipment, or to update patients' goals and progression, with no subtraction to the patients' treatment time.

From a qualitative point of view, the constraints imposed by the Q-Rehab algorithm establish explicit governance standards in planning the activities to be provided to patients, which can be constantly audited measuring the variance between the planned and delivered activities.

Staff feedback on Q-Rehab was similar to Primo, showing a relative easiness of use (about 70% of satisfaction) and a “usability” close to sufficient (SUS 65.8). The feedback on the usefulness (about 60% satisfaction) is difficult to interpret but can be related to different staff members using it in different ways (for example, for scheduling or reporting), hence giving different perspectives.

Other perceived barriers either for Priamo or Q-Rehab included the limited number of computers available to therapists and the need to use a different clinical software (on top of Priamo and Q-Rehab) not yet fully integrated. The continuous support by the Maugeri Rehab Processes team and the team champions has been a key enabler to the implementation of the DAIP.

The DAIP implementation was the result of a joint work between informatic engineers and the rehabilitation team, which required a long time for its development, a stepwise introduction until its active routine use in May 2020. Engineer support to the team has been available throughout as well as peer support, for junior members of the staff to be supported by the seniors. In fact, as by the 2019 OECD recommendation (32), the development of digital innovation should originate from a trust-based collaborative work with health professionals to ensure a conscious and lasting adoption of the technology.

No other publication is available to describe a digitally based model to support changes in an inpatient rehabilitation setting during the pandemic.

Our DAIP offers an example of how a DAIP infrastructure has supported our service in a very challenging period. Changes are difficult to be implemented at any time and place both at a personal and organizational level, leading always to a transitional destabilization and requiring a cultural shift. The pressure of the pandemic has added further chaos and complexity to the one that originates from any change occurring within an organization, although the sense of urge might have helped as a drive (33).

Our study presents many limitations. Most of all our observations range across a limited period of time, while changes are still occurring and the second wave of post-COVID-19 rehabilitation admissions might have not achieved its peak as yet.

The implication on patients' outcome has been addressed in general terms, with no reference to the outcome of the post-COVID-19 patients. However, it would have been difficult to interpret and compare the results to our studies due to the limited number of post-COVID-19 patients in our study and in lack of data in the literature about post-COVID-19 rehabilitation outcomes.

Although we have shown how the implementation of the DAIP system has served workflow efficiency, we have not specifically addressed the costs of the implementation as well as the potential savings.

Moreover, staff satisfaction was gathered from a percentage of the team members, and as such, it has not taken into account the collective perspective. A more in-depth understanding of the difficulties that staff members have encountered and the implications for their work and patients' treatment could be gathered by interviews or focus groups. Furthermore, we have not collected measures of patients' satisfaction about their admission and more specifically about the processes regarding their goal setting and the allocation of therapy sessions.

The measures of efficiency that we used present some limitations. First of all, we do not have these data for 2019 for comparison. Only the time spent for scheduling and recording therapy sessions was measured in both periods, manually in 2019 and by Q-Rehab in 2020, showing an increased efficiency due to the use of the software.

Considering the limited time of the observation and the limited volume of the staff–patient sample, we consider our findings to be preliminary and not generalizable or transferrable to other settings.

## Conclusion and Future Work

By applying the DAIP to rehabilitation treatment, it was shown that the management of rehabilitation can be efficiently performed even in the COVID-19 pandemic. This platform has served as a sound infrastructure for the team's work, allowing the rapid implementation of clinical and operational changes and facilitating the interaction between staff members from remote. As facts changed rapidly, we also had to adapt our minds to the changes, although likely with a slower pace and not without difficulties.

In particular, the DAIP provided a qualitative support for goal setting from remote together with an efficient way of planning and recording therapy sessions. Its implementation was generally well-perceived by the staff. Importantly, the processes supported by the DAIP can be easily audited against quality standards.

We envisage further developments of the DAIP during the COVID-19 and post-COVID-19 pandemic, starting from a more in-depth team feedback and from acquiring patients' feedback and considering how the time saved in certain processes could be reallocated to meet the needs still unmet and its implications on costs.

## Data Availability Statement

The raw data supporting the conclusions of this article will be made available by the authors, without undue reservation.

## Ethics Statement

Ethical review and approval was not required for the study on human participants in accordance with the local legislation and institutional requirements. Written informed consent was not provided because this study is based on retrospective data which are routinely collected by our Service. All patients admitted to our Service sign a consent to agree to the use of their personal data for research purposes.

## Author Contributions

All authors listed have made a substantial, direct and intellectual contribution to the work, and approved it for publication.

## Conflict of Interest

GP and GG declare their conflict of interest for their business involvement as data scientists at SurgiQ Srl and developers of Q-Rehab. The remaining authors declare that the research was conducted in the absence of any commercial or financial relationships that could be construed as a potential conflict of interest.
